# LncRNA ARAP1‐AS2 promotes high glucose‐induced human proximal tubular cell injury via persistent transactivation of the EGFR by interacting with ARAP1

**DOI:** 10.1111/jcmm.15897

**Published:** 2020-09-23

**Authors:** Xin Li, Tian‐Kui Ma, Si Wen, Lu‐Lu Li, Li Xu, Xin‐Wang Zhu, Cong‐Xiao Zhang, Nan Liu, Xu Wang, Qiu‐Ling Fan

**Affiliations:** ^1^ Department of Nephrology First Hospital of China Medical University Shenyang China; ^2^ Department of Clinical Laboratory First Hospital of China Medical University Shenyang China; ^3^ Department of Gastroenterology First Hospital of China Medical University Shenyang China

**Keywords:** diabetic nephropathy, EGFR/TGF‐β/Smad3 pathway, lncRNA ARAP1‐AS2, proximal tubular cell

## Abstract

The persistent transactivation of epidermal growth factor receptor (EGFR) causes subsequent activation of the TGF‐β/Smad3 pathway, which is closely associated with fibrosis and cell proliferation in diabetic nephropathy (DN), but the exact mechanism of persistent EGFR transactivation in DN remains unclear. ARAP1, a susceptibility gene for type 2 diabetes, can regulate the endocytosis and ubiquitination of membrane receptors, but the effect of ARAP1 and its natural antisense long non‐coding RNA (lncRNA), ARAP1‐AS2, on the ubiquitination of EGFR in DN is not clear. In this study, we verified that the expression of ARAP1 and ARAP1‐AS2 was significantly up‐regulated in high glucose‐induced human proximal tubular epithelial cells (HK‐2 cells). Moreover, we found that overexpression or knockdown of ARAP1‐AS2 could regulate fibrosis and HK‐2 cell proliferation through EGFR/TGF‐β/Smad3 signalling. RNA pulldown assays revealed that ARAP1‐AS2 directly interacts with ARAP1. Coimmunoprecipitation, dual‐immunofluorescence and ubiquitination assays showed that ARAP1 may maintain persistent EGFR activation by reducing EGFR ubiquitination through competing with Cbl for CIN85 binding. Taken together, our results suggest that the lncRNA ARAP1‐AS2 may promote high glucose‐induced proximal tubular cell injury via persistent EGFR/TGF‐β/Smad3 pathway activation by interacting with ARAP1.

## 1 INTRODUCTION

Currently, diabetic nephropathy (DN), the main cause of end‐stage renal disease (ESRD), is one of the most serious complications of diabetes.^1^ Studies have indicated that the development of renal fibrosis promotes the deterioration of kidney function in DN.^2^ Epidermal growth factor receptor (EGFR), a member of the ErbB family, has tyrosine kinase activity. EGFR transactivation is crucial for TGFβ/Smad3 activation and renal fibrosis in DN.^3^, ^4^, ^5^, ^6^ However, the mechanism of sustained EGFR activation in the progression of DN remains largely mysterious. Genome‐wide association studies (GWAS) have reported that the 11q13.4 locus near ARAP1 is significantly associated with type 2 diabetes.^7^ Studies in HeLa cells suggested that ARAP1 can regulate the internalization and ubiquitination of EGFR,^8^, ^9^ but the role of ARAP1 in regulating the ubiquitination of EGFR in DN has not been reported.

Long non‐coding RNAs (lncRNAs), defined as RNAs >200 nt in length that lack protein‐coding capability, participate in the renal fibrosis process in DN.^10^, ^11^, ^12^ By using the Arraystar human lncRNA/mRNA expression profile microarray (v3.0), we previously reported 21 up‐regulated lncRNAs, including the lncRNAs ARAP1‐AS2, and 726 up‐regulated mRNAs, including the mRNAs ARAP1 in the sera of DN patients compared with that of healthy controls.^13^ In this study, ARAP1‐AS2 and ARAP1 expression were significantly up‐regulated in HK‐2 cells cultured under high‐glucose conditions. We previously reported that up‐regulated ARAP1‐AS2 and ARAP1 may regulate the epithelial‐mesenchymal transition process and cytoskeleton rearrangement in HK‐2 cells under high‐glucose conditions by up‐regulating Cdc42‐GTP levels.^14^ This finding indicates that ARAP1‐AS2 and ARAP1 may participate in DN progression. However, to date, the functions of ARAP1‐AS2 and ARAP1 in the process of membrane receptor ubiquitination and their molecular mechanism in DN remain unclear.

In this study, we investigated whether ARAP1‐AS2 and ARAP1 participate in high glucose‐induced fibrosis and proximal tubular cell proliferation through regulating the ubiquitination of EGFR. Our results showed that ARAP1‐AS2 could regulate fibrosis and proximal tubular cell proliferation in DN by directly interacting with ARAP1 and maintaining persistent activation of the EGFR/TGF‐β/Smad3 pathway through reducing the ubiquitination of EGFR.

## 2 MATERIALS AND METHODS

### 2.1 Cell culture

Human proximal tubular epithelial cells (HK‐2 cells) were purchased from the American Type Culture Collection (ATCC) and cultured in medium (DMEM/F12, 1:1 mixture; Gibco) containing 10% foetal bovine serum, streptomycin (100 µg/mL, Gibco) and penicillin (100 U/mL, Gibco) in humidified air containing 5% CO_2_ at 37°C. The cells were randomly divided into two groups: a (1) normal‐glucose group (NG group), which was administered 5.5 mmol/L D‐glucose + 19.5 mmol/L mannitol and a (2) high‐glucose group (HG group), which was administered 25 mmol/L D‐glucose. Cells were exposed to high‐glucose conditions for 48 hours.

### 2.2 Plasmids, siRNAs, the EGFR tyrosine kinase inhibitor AG1478 and transfection

An ARAP1‐AS2 overexpression plasmid synthesized using the pcDNA3.1 expression vector was purchased from Sangon Biotechnology. The pLKO.1‐shRNA plasmid encoding shRNAs targeting human ARAP1 was synthesized and purchased from Vigene Biosciences. ARAP1‐AS2 siRNAs and scrambled siRNA were purchased from Sangon Biotechnology. The specific EGFR tyrosine kinase inhibitor AG1478 (MedChemExpress LLC) was used to suppress the phosphorylation of EGFR in HK‐2 cells. Powdered AG1478 was dissolved in DMSO, and the final concentration of AG1478 administered to HK‐2 cells was 10 μmol/L. Cells were exposed to AG1478 for 48 hours.

In the experiment, all plasmids were transfected at an amount of 3000 ng, and all siRNAs were transfected with jetPRIME^®^ at a 50 nmol/L final concentration following the manufacturer's protocol. HK‐2 cells in the HG group were transfected with ARAP1‐AS2 siRNA, the ARAP1‐AS2 overexpression plasmid and ARAP1 shRNA and named the HG + siARAP1‐AS2, HG + ARAP1‐AS2(+) and HG + shARAP1 groups, respectively. Cells in the HG group transfected with siRNANC targeting ARAP1‐AS2, the empty vector targeting ARAP1‐AS2, and shNC targeting ARAP1 were named the HG + siNC and HG + Vector and HG + shNC groups, respectively, and cells in the HG group that did not undergo transfection were named the HG mock group. HK‐2 cells in the NG group were transfected with ARAP1‐AS2 siRNA, the ARAP1‐AS2 overexpression plasmid and ARAP1 shRNA and named the NG + siARAP1‐AS2, NG + ARAP1‐AS2(+) and NG + shARAP1 groups, respectively. Cells in the NG group transfected with siRNANC targeting ARAP1‐AS2, the empty vector targeting ARAP1‐AS2, and shNC targeting ARAP1 were named the NG + siNC and NG + Vector and NG + shNC groups, respectively, and cells in the NG that did not undergo transfection were named the NG mock group. qRT‐PCR was used to detect the transfection efficacy.

### 2.3 Rapid amplification of cDNA 5′ and 3′ ends

The full‐length ARAP1‐AS2 sequence was obtained by rapid amplification of cDNA 5′ and 3′ ends (RACE) conducting with a GeneRacer™ kit (Invitrogen) following the manufacturer's instructions. Gene‐specific primer (GSP) sequences are provided in the supplementary material (Table S3).

### 2.4 Analysis of lncRNA coding potential

The coding potential of ARAP1‐AS2 was predicted by ORF finder (www.ncbi.nlm.nih.gov/orffinder/) and the Coding Potential Assessment Tool (CPAT) (http://lilab.research. bcm.edu/cpat/).

### 2.5 qRT‐PCR

Total RNA was extracted from the cells with TRIzol (Invitrogen) following the manufacturer's instructions. The RNA was reverse transcribed with the GoScript^TM^ Reverse Transcription System (Promega, USA). GoTaq® qPCR Master Mix (Promega, USA) was used for qPCR on a CFX96 PCR System (Bio‐Rad, Hercules, CA). Relative quantitative data regarding lncRNA and mRNA levels were analysed by the 2^‐ΔΔCt^ method and normalized to β‐actin levels. Primer sequences are provided in the supplementary material (Table S4).

### 2.6 Fluorescence in situ hybridization (FISH)

ARAP1‐AS2 FISH probes were synthesized by GenePharma, and the sequences are provided in the supplementary material (Table S5). All probes were labelled with Cy3. FISH was performed with a FISH kit following the manufacturer's instructions (GenePharma). All images were observed with a fluorescence microscope and analysed with LAS AF Lite (Leica).

### 2.7 RNA pulldown assay

Biotin‐labelled sense or antisense oligos of ARAP1‐AS2 were incubated with HK‐2 cell lysate for 1 hour. The complex was pulled down by streptavidin‐coated magnetic beads (M‐280 Dynabeads; Invitrogen). Sense and antisense probes were purchased from Sangon Biotechnology, and their sequences are provided in the supplementary material (Table S6). The amount of ARAP1 mRNA was measured by qRT‐PCR.

### 2.8 RNA pulldown sequencing (pulldown‐seq)

RNA pulldown‐enriched RNA was used to construct a library suitable for the Illumina high‐throughput sequencing platform with the NEBNext Ultra II RNA Library Prep | NEB library construction kit. After RNA isolation, enriched and purified RNA underwent RNA fragmentation, double‐stranded DNA synthesis, end repair, linker ligation, ligation product purification, fragment size sorting, and library amplification processes to obtain libraries of specific lengths, and sequencing libraries were subjected to strict quality control to improve library stability and repeatability. HTSeq was used to calculate the FPKM value, and we analysed the difference between input and pulldown samples to identify lncRNA‐bound mRNAs.

### 2.9 Western blot analysis

Cells were lysed in RIPA buffer containing phenylmethanesulfonylfluoride. Proteins were separated by SDS‐PAGE and transferred onto PVDF membranes. The blots were incubated with primary antibody overnight at 4°C, followed by incubation with HRP‐conjugated secondary antibody. Signals were visualized using ECL substrates (Millipore). The antibody dilution ratios are provided in the supplementary material (Table S7). β‐Actin served as an internal control. The grey value of the protein bands was analysed by ImageJ software.

### 2.10 Cell Counting Kit‐8 assay

Cells were seeded in a 96‐well plate at 3 × 10^3^ cells per well and incubated overnight. On days 1, 2 and 3, 10 µL of CCK‐8 reagent (Dojindo) was added to the culture medium of each well, and the 96‐well plate was placed in an incubator and maintained at 37°C for 2 hours. Then, a microplate reader (BioTek Instruments) was used to measure absorbance.

### 2.11 Coimmunoprecipitation

Coimmunoprecipitation experiments were performed as previously described.^15^ The primary antibodies used were as follows: anti‐ARAP1 (sc‐367892, Santa Cruz Biotechnology), anti‐CIN85 (sc‐166862, Santa Cruz Biotechnology), and anti‐IgG (ab172730, Abcam).

### 2.12 Ubiquitination assay

NG and HG cells were transfected with shARAP1 or empty vector. After transfection, the cells were treated with the proteasome inhibitor MG132 at 50 μg/mL (MedChemExpress LLC) for 12 hours. The cells were then lysed in co‐IP lysis buffer (Beyotime) and immunoprecipitated with anti‐EGFR antibody overnight at 4°C. Anti‐ubiquitin antibody (#3933, Cell Signaling Technology) was used to detect ubiquitinated EGFR in the precipitates. The precipitates and lysates were analysed by Western blotting to detect the protein expression of ARAP1, EGFR and β‐actin.

### 2.13 Animals

The db/db mouse, which has genetic defects in the leptin receptor, is a well‐established model for type 2 diabetes, obesity and insulin resistance. Eight‐week‐old diabetic male db/db mice (C57BLKS/J‐lepr db/leprdb, n = 10, DN group) and non‐diabetic male db/m mice (C57BLKS/J‐leprdb/+, n = 10, normal group) were purchased from the Model Animal Research Center of Nanjing University (Nanjing, China). The animals were housed at 23 ± 3°C under a 12‐hours light/dark cycle under 50% ± 20% humidity at the Laboratory Animal Center of China Medical University and given free access to water and food. The body weight, urinary albumin/creatinine ratio and blood glucose in 20‐week‐old db/db mice were significantly higher than those in db/m mice at the same age (Figure S3). Renal cortical tissue was harvested for subsequent studies at 20 weeks of age. All experimental methods were approved by the Institutional Animal Care and Use Committee of China Medical University, and ethical approval was obtained from the Ethics Committee of China Medical University (number: CMU2019222).

### 2.14 Clinical renal biopsy samples

Renal tissue was obtained at the First Hospital of China Medical University. Normal kidney tissue was obtained from nephrectomies performed for tumours, and DN tissue was obtained from renal biopsies of adults with DN. The renal tissue specimens were used in accordance with the ethical standards of the First Hospital of China Medical University (number: AF‐SOP‐07‐1.0‐01) and the Declaration of Helsinki of 1975, as revised in 1983. At hospital admission, all patients consented to experiments on their anonymized archived tissue samples.

### 2.15 Immunofluorescence staining

#### 2.15.1 For HK‐2 cells

Cells cultured on coverslips with normal or high levels of glucose were fixed with 4% paraformaldehyde and incubated with 0.1% Triton X to permeabilize the cell membrane. The cells were then blocked with goat serum and incubated with primary antibody at 4°C overnight. The cells were then washed with PBS three times and incubated with fluorescent label‐conjugated secondary antibody at room temperature. Nuclei were stained with DAPI. All images were observed with a fluorescence microscope and analysed with LAS AF Lite (Leica). The primary antibodies used were mouse anti‐ARAP1 (1:50, sc‐393138, Santa Cruz Biotechnology) and rabbit anti‐CIN85 (1:250, ab151574, Abcam).

#### 2.15.2 For tissue sections

Renal tissues were fixed and embedded in paraffin and used to prepare 3‐μm sections from mouse tissues and 1.5‐μm sections from human tissues. The sections were deparaffinized and heated in sodium citrate buffer (0.01 mol/L, pH 6.0). Then, the sections were cooled at room temperature and washed with PBS three times. For immunofluorescence staining, the tissues were then dissolved with 0.1% Triton X, blocked in goat serum and subsequently incubated with primary antibody at 4°C overnight. The sections were washed and incubated with fluorescent label‐conjugated secondary antibody at room temperature. Nuclei were stained with DAPI. All images were observed with a fluorescence microscope and analysed with LAS AF Lite (Leica). The primary antibodies used were mouse anti‐ARAP1 (1:50, sc‐393138, Santa Cruz Biotechnology) and rabbit anti‐CIN85 (1:100, ab151574, Abcam).

### 2.16 Statistical analyses

We used SPSS 26.0 software (IBM) to perform statistical analyses of the data. Data are expressed as the mean ± SD of at least three separate experiments. Student's *t* test and one‐way analysis of variance (no fewer than three groups) were used to analyse the significance of differences between groups. *P* < .05 indicated a statistically significant difference. GraphPad Prism 7.0 software (GraphPad software) was used to conduct statistical analyses.

## 3 RESULT

### 3.1 ARAP1‐AS2 was significantly up‐regulated in HK‐2 cells cultured under high glucose and mainly distributed in the nuclei of HK‐2 cells

**Figure 1:**
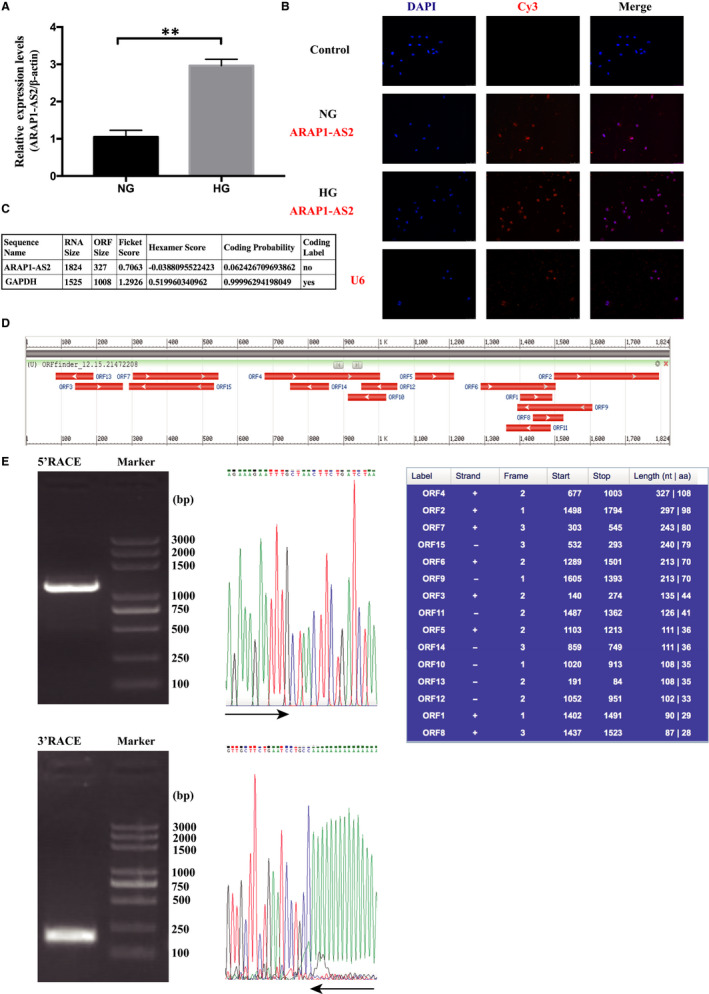
The expression and distribution of ARAP1‐AS2 in HK‐2 cells. A, qRT‐PCR analysis of the expression of ARAP1‐AS2 in HK‐2 cells of the normal‐glucose (NG) group and high‐glucose (HG) group (n = 3); the data are representative of three independent experiments. Data are presented as the mean ± SD. *P < .05, **P < .01, NS, no significant difference. B, Localization of ARAP1‐AS2 (red) in HK‐2 cells that were stimulated by high or normal glucose determined by FISH. ARAP1‐AS2 was mainly distributed in the cell nuclei (×400). Bar = 50 μmol/L. All probes were labelled with Cy3. HK‐2 cells in the control group did not exhibit self‐illumination. C, Coding Potential Assessment Tool (CPAT) was used to test the protein‐coding ability of ARAP1‐AS2. D, ORF finder was used to analyse the protein‐coding ability of ARAP1‐AS2. E, 5′ and 3′ rapid amplification of cDNA ends (RACE) assays in HK‐2 cells were used to detect the whole sequence of ARAP1‐AS2. Left: an image of PCR products from the 5′‐RACE and 3′‐RACE assays separated by gel electrophoresis. Right: sequencing of PCR products indicated the boundary between the universal anchor primer and ARAP1‐AS2 sequences

The abnormal expression of ARAP1‐AS2 in the sera of DN patients (data not shown) was reported in our previous study.^13^ We further verified the expression of ARAP1‐AS2 in HK‐2 cells stimulated with high glucose by qRT‐PCR and found that ARAP1‐AS2 was significantly up‐regulated in the HG group compared with the NG group (Figure 1A). The results of FISH (Figure 1B) revealed that in the NG and HG groups, ARAP1‐AS2 was expressed in both the nucleus and cytoplasm, but ARAP1‐AS2 was mainly expressed in the nucleus. In addition, CPAT and ORF finder were used to predict the protein‐coding ability of ARAP1‐AS2, and the amino acid sequences predicted by ORF finder were compared in NCBI (Figure 1C,D). The results showed that the coding probability of ARAP1‐AS2 was 0.06, suggesting that ARAP1‐AS2 has almost no coding capability. The full‐length ARAP1‐AS2 sequence was successfully obtained by RACE (Figure 1E and Figure S1). In summary, all of the above results revealed that ARAP1‐AS2 may play a role in the nuclei of HK‐2 cells.

### 3.2 ARAP1‐AS2 promoted HK‐2 cell proliferation and fibrosis

**Figure 2:**
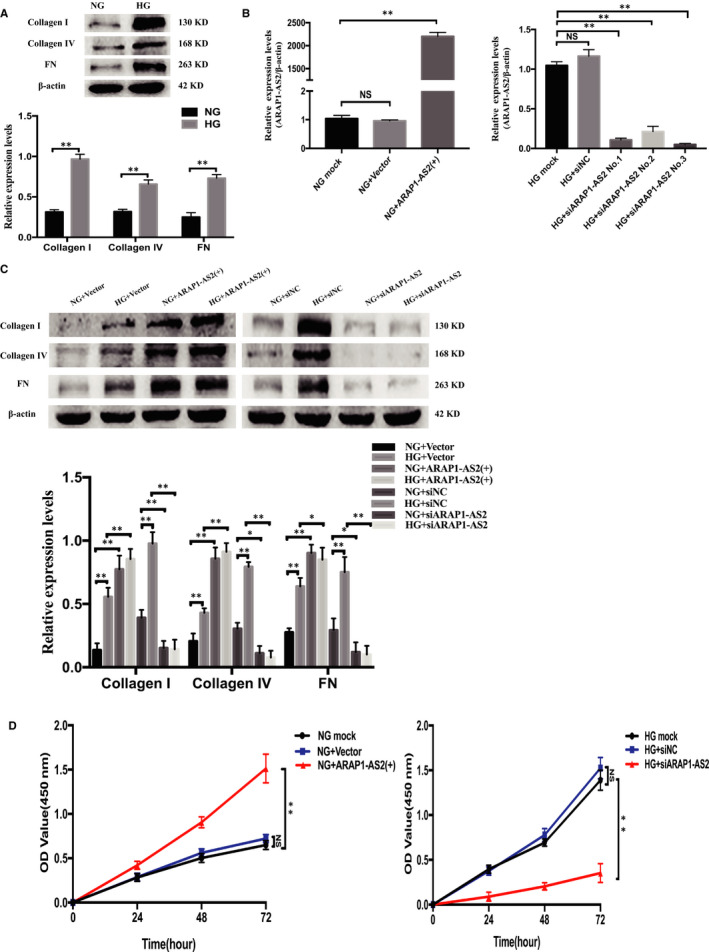
ARAP1‐AS2 regulated fibrosis and HK‐2 cell proliferation. A, The protein expression levels of collagen I, collagen IV and fibronectin (FN) in HK‐2 cells of the normal‐glucose (NG) group and high‐glucose (HG) group (n = 3) were detected by Western blot analysis. B, Forty‐eight hours after transfection of the ARAP1‐AS2 overexpression plasmid (3000 ng) in the NG group or ARAP1‐AS2 siRNAs (50 nmol/L) in the HG group in 6‐well plates, ARAP1‐AS2 overexpression and knockdown efficiencies were assessed by qRT‐PCR. C, Forty‐eight hours after transfection of the ARAP1‐AS2 overexpression plasmid or ARAP1‐AS2 siRNA in the NG group and HG group, the protein expression levels of collagen I, collagen IV and FN were detected by Western blot analysis. D, After transfection of the ARAP1‐AS2 overexpression plasmid or ARAP1‐AS2 siRNA in the NG group and HG group, cell proliferation was detected by CCK‐8 analysis at 24, 48 and 72 h. In all panels, the data are representative of three independent experiments. Data are presented as the mean ± SD. *P < .05, **P < .01, NS, not significant

The results of Western blot analysis showed that expression of the matrix proteins collagen I, collagen IV and fibronectin (FN), was significantly increased in the HG group compared with the NG group (Figure 2A). We next confirmed that the ARAP1‐AS2 overexpression plasmid, which contained the full‐length ARAP1‐AS2 sequence obtained from RACE, could enhance the expression of ARAP1‐AS2, and qRT‐PCR showed that siARAP1‐AS2(No. 3) had the best knockdown efficacy (Figure 2B). The siRNA sequence targeting ARAP1‐AS2 is provided in the supplementary material (Table S2). The ARAP1‐AS2 overexpression plasmid was also confirmed through gel electrophoresis and sequencing after restriction enzyme digestion (Figure S2). The results of Western blot analysis indicated that the expression levels of collagen I, collagen IV and FN were significantly increased by the ARAP1‐AS2 overexpression plasmid in both the NG and HG groups, while their expression was decreased by siARAP1‐AS2 transfection in both the NG and HG groups (Figure 2C). Moreover, the CCK‐8 assay revealed that cell proliferation was significantly increased in the NG + ARAP1‐AS2 (+) group compared with the NG + Vector and NG mock groups. Furthermore, cell proliferation was significantly inhibited in the HG + siARAP1‐AS2 group compared with the HG + siNC and HG mock groups (Figure 2D). These data suggested that ARAP1‐AS2 could regulate fibrosis and cell proliferation in HK‐2 cells.

### 3.3 ARAP1‐AS2 directly interacts with ARAP1 and controls ARAP1 mRNA and protein expression

**Figure 3:**
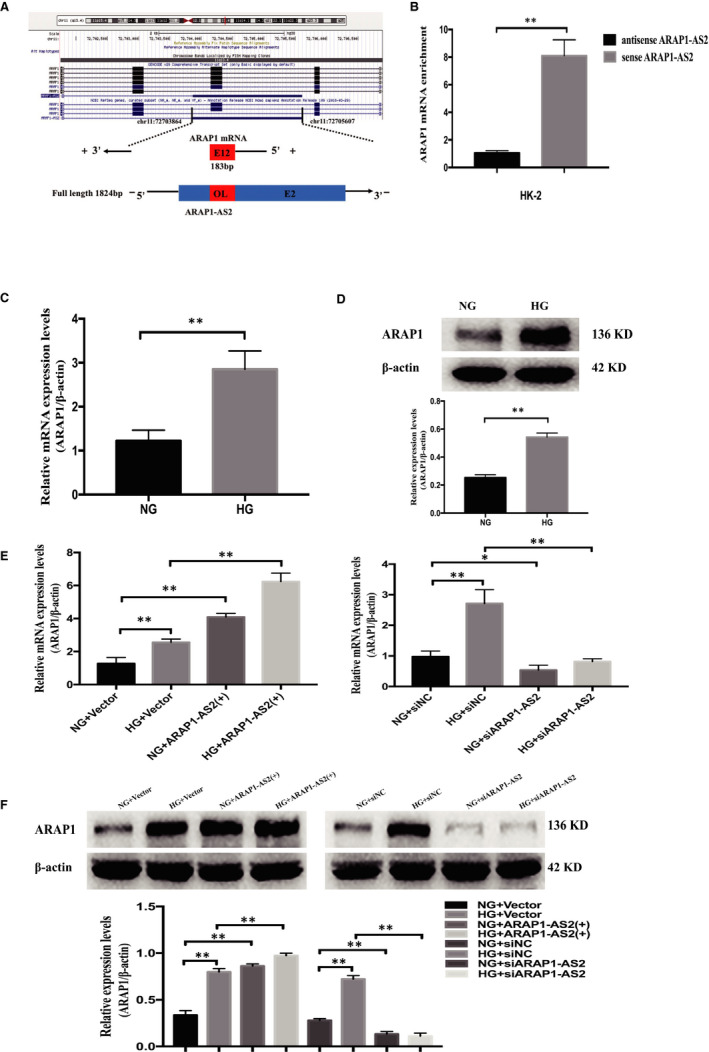
ARAP1‐AS2 interacts with ARAP1. A, The 12th exon of ARAP1 (which is 183 bp in length) overlaps the second exon of ARAP1‐AS2. B, RNA pulldown assays were performed to detect whether ARAP1‐AS2 could bind ARAP1 in HK‐2 cells. ARAP1 was pulled down by ARAP1‐AS2, and the level of ARAP1 was detected by qRT‐PCR. C, The mRNA expression levels of ARAP1 in HK‐2 cells of the normal‐glucose (NG) group and high‐glucose (HG) group (n = 3) were detected by qRT‐PCR. D, The protein expression levels of ARAP1 in HK‐2 cells of the NG group and HG group (n = 3) were detected by Western blot analysis. E, Forty‐eight hours after transfection of the ARAP1‐AS2 overexpression plasmid or ARAP1‐AS2 siRNA in the NG group and HG group, the mRNA expression level of ARAP1 was detected by qRT‐PCR. F, Forty‐eight hours after transfection of the ARAP1‐AS2 overexpression plasmid or ARAP1‐AS2 siRNA in the NG group and HG group, the protein expression level of ARAP1 was detected by Western blot analysis. In all panels, the data are representative of three independent experiments. Data are presented as the mean ± SD. *P < .05, **P < .01, NS, not significant

Antisense lncRNAs can regulate the expression of downstream genes by directly binding sense mRNA.^16^ By bioinformatics and gene sequence analyses, we found that the 12th exon of ARAP1, the full length of which is 183 bp, overlaps the second exon of ARAP1‐AS2 (Figure 3A), which may provide a structural foundation for the regulatory relationship between these two molecules. We therefore applied biotin‐labelled RNA pulldown assays to detect whether ARAP1‐AS2 could pull down ARAP1 in HK‐2 cells. The data indicated that ARAP1 was pulled down by sense ARAP1‐AS2; however, antisense ARAP1‐AS2 was unable to pull down ARAP1 (Figure 3B). These results suggest that ARAP1‐AS2 could directly interact with ARAP1 in HK‐2 cells. We further verified the expression of ARAP1 in HK‐2 cells cultured under high‐glucose or normal‐glucose conditions by qRT‐PCR and Western blot analysis, which showed that the mRNA and protein expression of ARAP1 was significantly increased in the HG group compared with the NG groups (Figure 3C,D). ARAP1‐AS2 could regulate the expression of ARAP1. The results of qRT‐PCR and Western blot analysis revealed that the levels of ARAP1 were significantly up‐regulated by ARAP1‐AS2 (+) in both the NG and HG groups, while the expression of ARAP1 was decreased by siARAP1‐AS2 in both the NG and HG groups (Figure 3E,F). Taken together, these results suggest that ARAP1‐AS2 may play a role in high glucose‐induced HK‐2 cells by interacting with ARAP1.

### 3.4 ARAP1 interacts with CIN85 and regulates the ubiquitination of EGFR

**Figure 4:**
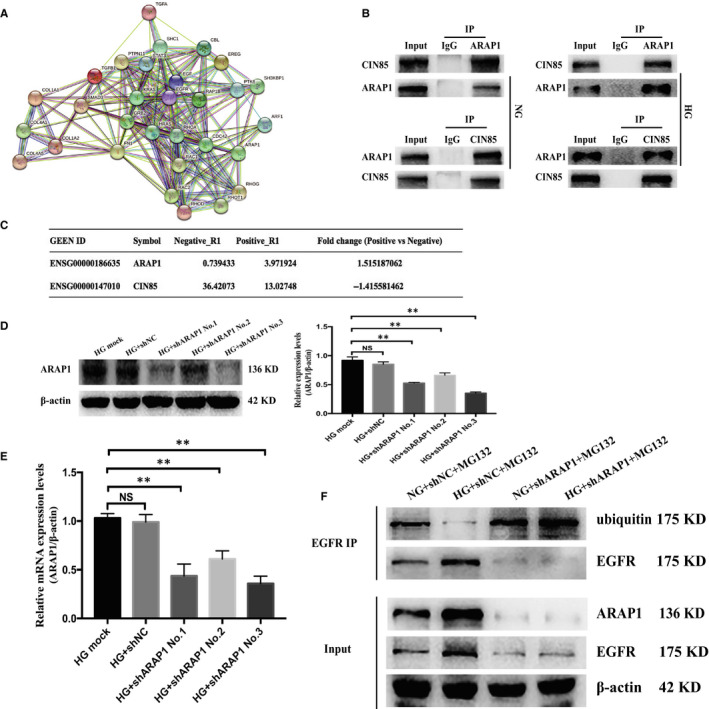
ARAP1 interacts with CIN85 and regulates the ubiquitination of EGFR. A, Bioinformatic analysis of protein‐protein interaction networks by STRING v.10. A screenshot from STRING shows a network associated with ARAP1 and CIN85 and EGFR. B, The physical interaction between ARAP1 and CIN85 was detected by coimmunoprecipitation analysis. IgG was used as a negative control. C, RNA pulldown‐seq of ARAP1‐AS2. Fold change value = Log_2_((FPKM in Positive + 1)/(FPKM in Negative + 1)). A higher fold change value indicates more enrichment. D, Forty‐eight hours after transfection of ARAP1 shRNA (3000 ng) in the HG group in 6‐well plates, the ARAP1 knockdown efficiency was detected by Western blot analysis. E, Forty‐eight hours after transfection of ARAP1 shRNA (3000 ng) in the HG group in 6‐well plates, ARAP1 knockdown efficiency was assessed by qRT‐PCR. F, ARAP1 knockdown increased EGFR ubiquitination in the NG group and HG group. In all panels, the data are representative of three independent experiments. Data are presented as the mean ± SD. *P < .05, **P < .01, NS, not significant

**Figure 5:**
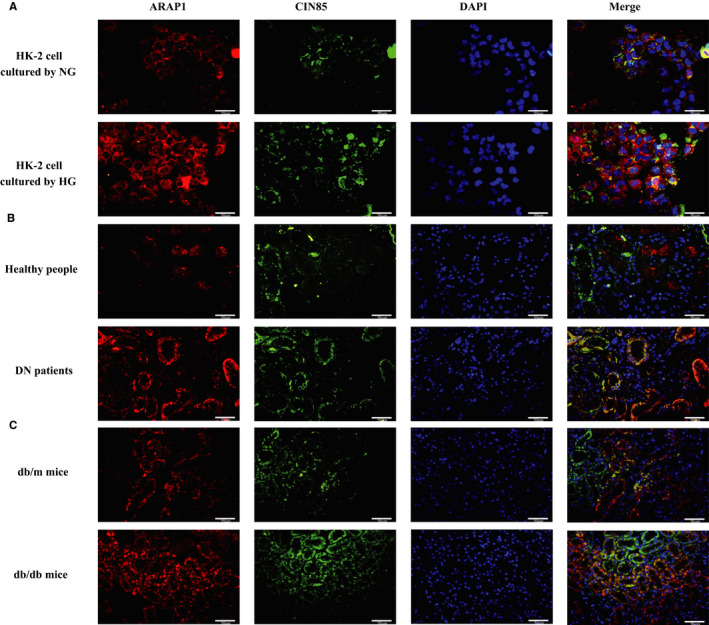
Dual immunofluorescent staining for ARAP1 and CIN85. A, Dual immunofluorescent staining for ARAP1 and CIN85 in HK‐2 cells of the normal‐glucose (NG) group and high‐glucose (HG) group (×400). Bar = 50 μmol/L. B, Dual immunofluorescent staining for ARAP1 and CIN85 in the renal tissues of healthy control and DN patients (×400). Bar = 50 μmol/L. C, Dual immunofluorescent staining for ARAP1 and CIN85 in the renal tissues of normal control and db/db mice (×400). Bar = 50 μmol/L

CIN85 regulates the ubiquitination and internalization of EGFR by forming the Cbl–CIN85–endophilin complex.^17^ In addition, ARAP1 was reported to compete with Cbl for CIN85 binding in early endosomes and regulate the ubiquitination of EGFR in HeLa cells.^8^, ^18^ We further studied the potential molecular mechanism of the relationship between ARAP1 and EGFR in HK‐2 cells. STRING v.10 predicted a protein‐protein interaction network associated with ARAP1, EGFR and CIN85, which suggests that ARAP1 might interact with CIN85 and then affect EGFR (Figure 4A). Moreover, coimmunoprecipitation assays demonstrated the interaction between ARAP1 and CIN85 in HK‐2 cells (Figure 4B). The RNA pulldown‐seq results showed that the complexes pulled down by ARAP1‐AS2 were not enriched in CIN85 but were enriched in ARAP1 (Figure 4C). This result revealed that ARAP1‐AS2 may regulate CIN85 indirectly through interacting with ARAP1. Western blot analysis and qRT‐PCR showed that shARAP1 (No. 3) had the best knockdown effect (Figure 4D,E); therefore, we chose shARAP1 (No. 3) for ARAP1 knockdown in this study. The shRNA sequence targeting ARAP1 is provided in the supplementary material (Table S1). ARAP1 knockdown increased the level of EGFR ubiquitination in both the NG and HG groups (Figure 4F). Moreover, the results of dual‐immunofluorescence staining showed that the colocalization between ARAP1 and CIN85 was increased in high glucose‐induced HK‐2 cells (Figure 5A), the renal tissues of DN patients (Figure 5B) and db/db mice (Figure 5C) compared with that in the corresponding controls. Collectively, these findings suggest that ARAP1 may regulate the ubiquitination of EGFR in DN by interacting with CIN85.

### 3.5 ARAP1 promoted the fibrosis and proliferation of HK‐2 cells in DN by maintaining the persistent activation of EGFR and subsequent activation of the TGF‐β/Smad3 pathway

**Figure 6:**
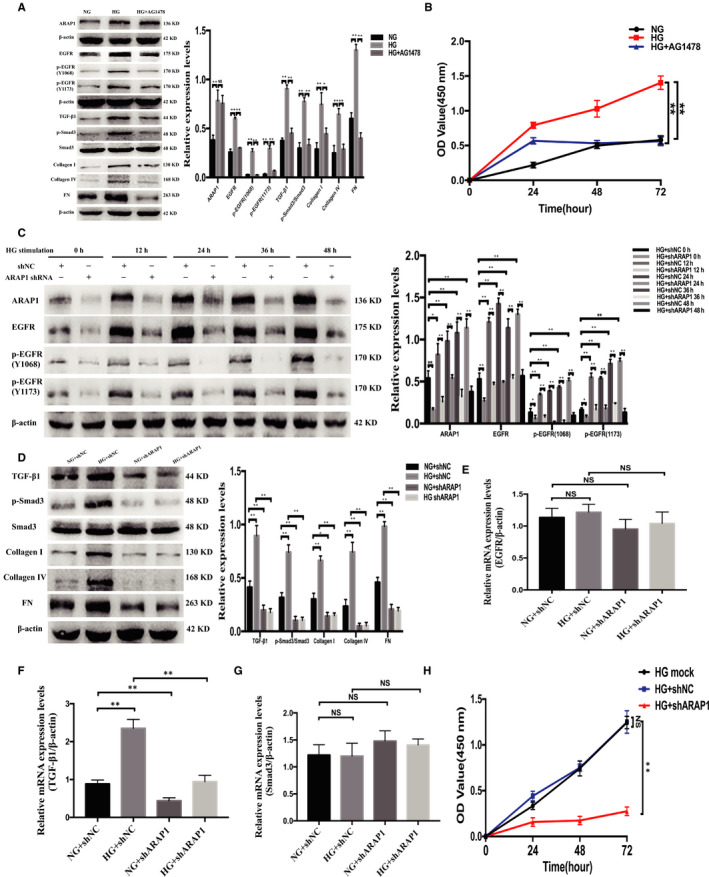
ARAP1 regulates activation of the EGFR/TGF‐β/Smad3 pathway in HK‐2 cells. A, The protein expression levels of ARAP1, total EGFR, EGFR phosphorylated at two phosphorylation sites (Y1068 and Y1173), TGF‐β1, p‐Smad3, collagen I, collagen IV and fibronectin (FN) in the normal‐glucose (NG) group, high‐glucose (HG) group and HG group administered the EGFR tyrosine kinase inhibitor AG1478 were detected by Western blot analysis. B, After the addition of ag1478 to the hg group, cell proliferation was detected by cck‐8 assay at 24, 48 and 72 h. C, Hk‐2 cells were pre‐treated with arap1 shrna for 24 h and then divided into the ng group and hg group. The protein expression levels of ARAP1, total EGFR, and EGFR phosphorylated at two phosphorylation sites (Y1068 and Y1173) were detected at 12, 24, 36 and 48 h after stimulation by high glucose. 0 h means that the HK‐2 cells were only transfected with ARAP1 shRNA for 24 h under normal‐glucose and were not stimulated with high glucose. High‐glucose stimulation was carried out for 12‐48 h. D, Forty‐eight hours after transfection of ARAP1 shRNA in the NG group and HG group, the protein expression of TGF‐β1, p‐Smad3, collagen I, collagen IV and FN was detected by Western blot analysis. E, Forty‐eight hours after transfection of ARAP1 shRNA in the NG group and HG group, the mRNA expression of EGFR was detected by qRT‐PCR. F, Forty‐eight hours after transfection of ARAP1 shRNA in the NG group and HG group, the mRNA expression of TGF‐β1 was detected by qRT‐PCR. G,. Forty‐eight hours after transfection of ARAP1 shRNA in the NG group and HG group, the mRNA expression of Smad3 was detected by qRT‐PCR. H, After transfection of ARAP1 shRNA in the HG group, cell proliferation was detected by CCK‐8 assay at 24, 48 and 72 h. In all panels, the data are representative of three independent experiments. Data are presented as the mean ± SD. *P < .05, **P < .01, NS, not significant

The TGF‐β1/Smad3 pathway was reported to be a pivotal pathway for the progression of renal fibrosis.^19^ Furthermore, persistent EGFR activation is critical for mediating sustained activation of the TGF‐β/Smad3 pathway in renal fibrosis.^3^ To investigate the effect of ARAP1 on the maintenance of persistent EGFR activation via the TGF‐β/Smad3 pathway in high glucose‐cultured HK‐2 cells, we examined the expression levels of ARAP1, total EGFR, EGFR phosphorylated at two phosphorylation sites (Y1068 and Y1173), TGF‐β1, p‐Smad3, collagen I, collagen IV and FN and found them to be increased in the HG group; furthermore, increases in the expression of all of these proteins (except ARAP1) could be inhibited by the specific EGFR tyrosine kinase inhibitor AG1478 (Figure 6A). Moreover, the CCK‐8 assay showed that HK‐2 cell proliferation in the HG group was significantly increased compared with that in the NG group and that this increase in proliferation was markedly decreased by AG1478 (Figure 6B). HK‐2 cells were pre‐treated with ARAP1 shRNA for 24 hours and then divided into the NG and HG groups. 0 hour means that the HK‐2 cells were only transfected with ARAP1 shRNA for 24 hours under normal‐glucose and were not stimulated with high glucose. Then, we collected cells at 12, 24, 36 and 48 hours after stimulation with high glucose to detect the protein expression levels of ARAP1, total EGFR and EGFR phosphorylated at two phosphorylation sites (Y1068 and Y1173). The Western blot results showed that the expression levels of ARAP1 and total EGFR and activation of EGFR due to its phosphorylation at two phosphorylation sites (Y1068 and Y1173) were persistently inhibited after shARAP1 transfection (Figure 6C). The expression of TGF‐β1, p‐Smad3, collagen I, collagen IV and FN were significantly decreased by shARAP1 in both the NG and HG groups (Figure 6D). The mRNA expression of TGF‐β1 was markedly inhibited by shARAP1 in both the NG and HG groups, but there was no significant change in the mRNA expression of EGFR and Smad3 after transfection with shARAP1 (Figure 6E‐G). In addition, the CCK‐8 assay revealed that cell proliferation in the HG + shARAP1 group was significantly decreased compared with that in the HG + shNC and HG mock groups (Figure 6H). These results suggest that ARAP1 may play a role in maintaining the persistent activation of EGFR and promoting fibrosis and cell proliferation via TGF‐β/Smad3 in high glucose‐induced HK‐2 cells.

### 3.6 ARAP1‐AS2 regulated high glucose‐induced HK‐2 cell proliferation and fibrosis by interacting with ARAP1 and subsequently activating the EGFR and TGF‐β/Smad3 pathways

**Figure 7:**
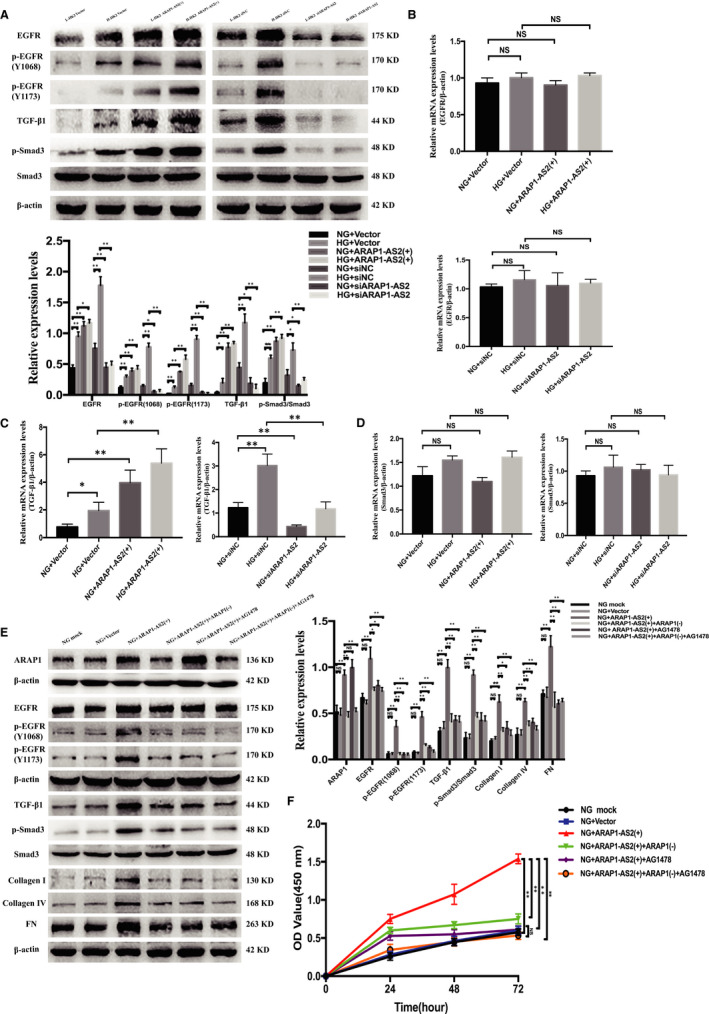
ARAP1‐AS2 promotes the expression of fibrosis and cell proliferation via the EGFR/TGF‐β/Smad3 pathway. A, Forty‐eight hours after transfection of the ARAP1‐AS2 overexpression plasmid or ARAP1‐AS2 siRNA in the normal‐glucose (NG) group and high‐glucose (HG) group, the protein expression levels of total EGFR, EGFR phosphorylated at two phosphorylation sites (Y1068 and Y1173), TGF‐β1 and p‐Smad3 were detected by Western blot analysis. B, Forty‐eight hours after transfection of the ARAP1‐AS2 overexpression plasmid or ARAP1‐AS2 siRNA in the NG group and HG group, the mRNA expression level of total EGFR was detected by qRT‐PCR. C, Forty‐eight hours after transfection of the ARAP1‐AS2 overexpression plasmid or ARAP1‐AS2 siRNA in the NG group and HG group, the mRNA expression level of TGF‐β1 was detected by qRT‐PCR. D, Forty‐eight hours after transfection of the ARAP1‐AS2 overexpression plasmid or ARAP1‐AS2 siRNA in the NG group and HG group, the mRNA expression level of Smad3 was detected by qRT‐PCR. E, Forty‐eight hours after cotransfection of the ARAP1‐AS2 overexpression plasmid and ARAP1 shRNA or transfection of the ARAP1‐AS2 overexpression plasmid accompanied by treatment with the EGFR tyrosine kinase inhibitor AG1478 in the NG group, the protein expression levels of ARAP1, total EGFR, EGFR phosphorylated at two phosphorylation sites (Y1068 and Y1173), TGF‐β1, p‐Smad3, collagen I, collagen IV and fibronectin (FN) were detected by Western blot analysis. F, After cotransfection of the ARAP1‐AS2 overexpression plasmid and ARAP1 shRNA or transfection of ARAP1‐AS2 overexpression plasmid accompanied by AG1478 treatment in the NG group, cell proliferation was detected by CCK‐8 assay at 24, 48 and 72 h. In all panels, the data are representative of three independent experiments. Data are presented as the mean ± SD. *P < .05, **P < .01, NS, not significant

Based on the interaction between ARAP1‐AS2 and ARAP1, we further investigated the effect of ARAP1‐AS2 on the EGFR/TGF‐β/Smad3 pathway in the NG and HG groups. Western blot analysis showed that ARAP1‐AS2 overexpression increased the levels of total EGFR, EGFR phosphorylated at two phosphorylation sites (Y1068 and Y1173), TGF‐β1 and p‐Smad3, while ARAP1‐AS2 knockdown significantly inhibited the levels of total EGFR, EGFR phosphorylated at two phosphorylation sites (Y1068 and Y1173), TGF‐β1 and p‐Smad3 (Figure 7A). The results of qRT‐PCR showed that the mRNA expression of TGF‐β1, but not EGFR or Smad3, was regulated by ARAP1‐AS2 (Figure 7B‐D).

To clarify the relationship between ARAP1‐AS2, ARAP1 and the EGFR/TGF‐β/Smad3 pathway, we further performed a rescue experiment in HK‐2 cells to explore the mechanism by which ARAP1‐AS2 regulates cellular proliferation and fibrosis. Western blot analysis showed that the effect of ARAP1‐AS2 overexpression in increasing the levels of ARAP1, total EGFR, EGFR phosphorylated at two phosphorylation sites (Y1068 and Y1173), TGF‐β1, p‐Smad3, collagen I, collagen IV and FN in the NG group was inhibited by APAP1 knockdown, while the increased expression of these proteins was also blocked by the EGFR tyrosine kinase inhibitor AG1478 but not ARAP1 (Figure 7E). Moreover, the CCK‐8 assay revealed that the effect of ARAP1‐AS2 overexpression in promoting cell proliferation in the NG group was blocked by APAP1 knockdown and AG1478 (Figure 7F). These results demonstrate that ARAP1‐AS2 may have regulated proximal tubular cell proliferation and fibrosis in high glucose‐induced HK‐2 cells by interacting with ARAP1 and subsequently activating the EGFR/TGF‐β/Smad3 pathway.

## 4 DISCUSSION

Many studies have reported that lncRNAs participate in DN through regulating target gene expression.^20^, ^21^ For example, silencing of the lncRNA XIST contributed to preventing renal interstitial fibrosis in DN.^22^ Ge et al^23^ also found that overexpression of the lncRNA NR_038323 regulated renal fibrosis via the miR‐324‐3p/DUSP1 axis in DN. Therefore, lncRNAs may play a role in the progression of DN, but their clear molecular mechanism in DN remains mysterious. In the current study, we found that ARAP1‐AS2 and ARAP1 were significantly increased in HK‐2 cells cultured under high‐glucose conditions, and ARAP1‐AS2 directly interacted with ARAP1 and then regulated the EGFR/TGF‐β/Smad3 pathway to promote high glucose‐induced proximal tubular cell injury. In addition, our previous study showed that the expression of ARAP1‐AS2 and ARAP1 was up‐regulated in the sera of DN patients.^13^ Therefore, these results suggest that ARAP1‐AS2 and ARAP1 may participate in the pathogenesis of DN.

ARAP1‐AS2 is a natural antisense lncRNA located on chromosome 11 (Chr11: 72700474‐72705607, 1824 bp). However, to date, there have been no reports on the mechanism of ARAP1‐AS2 in diseases. Natural antisense lncRNAs play important roles in many pathophysiological processes by regulating their sense gene expression at the transcriptional and posttranscriptional levels.^24^, ^25^ In the present study, we found that ARAP1‐AS2 is mainly a nuclear lncRNA in HK‐2 cells, implying its function in transcription and chromatin remodelling.^26^, ^27^ We further confirmed that ARAP1‐AS2 directly interacts with ARAP1 by RNA pulldown assay. Moreover, our study revealed that ARAP1‐AS2 overexpression could significantly promote proximal tubular cell proliferation and fibrosis, and the silencing of ARAP1‐AS2 led to the remarkable inhibition of HK‐2 cell proliferation and fibrosis induced by high glucose. Up‐regulated ARAP1‐AS2 may contribute to proximal tubular cell injury in DN by interacting with ARAP1.

ARAP1, single nucleotide polymorphisms (rs1552224 and rs11603334) in which were confirmed to increase type 2 diabetes susceptibility,^28^ connects phosphatidyl inositol 3 phosphate (PIP3) and phosphotyrosine signalling and the Arf and Rho signalling pathways.^29^ ARAP1 is mainly distributed at the plasma membrane, Golgi complex and endosomal compartments and can regulate the internalization and ubiquitination of EGFR.^8^, ^9^ High glucose can stimulate EGFR transactivation, which has been reported to occur in the pathogenesis of renal fibrosis in DN via the ERK, STAT3, AKT and TGF‐β/Smad3 pathways^4^, ^5^, ^6^, ^30^; however, the mechanism of sustained EGFR activation is unclear. The ubiquitin ligase (E3) Cbl and adaptor protein CIN85 (also known as SH3KBP1) are important regulators of EGFR internalization and degradation.^31^, ^32^ CIN85 could bind Cbl and recruit endocytic machinery components, such as endophilins, subsequently indirectly regulating the endocytosis of EGFR,^17^, ^33^ while ARAP1 could regulate the internalization and ubiquitination of EGFR by competing with Cbl for CIN85 binding in early endosomes in HeLa cells.^8^ The current study revealed that ARAP1 interacts with CIN85 under both high‐glucose and normal‐glucose conditions, and the colocalization between ARAP1 and CIN85 was increased in high glucose‐induced HK‐2 cells and the renal tissues of DN patients and db/db mice. RNA pulldown‐seq results showed that complexes pulled down via ARAP1‐AS2 were not enriched in CIN85 but were enriched in ARAP1. This may reveal that ARAP1‐AS2 may interact with ARAP1 and then regulate CIN85 indirectly. ARAP1 knockdown increased the ubiquitination of EGFR in HK‐2 cells, but qRT‐PCR results showed that ARAP1‐AS2 and ARAP1 could not regulate EGFR transcription. Moreover, our results suggest that persistent inhibition of ARAP1 maintained the decreased levels of total EGFR and the persistent inhibition of EGFR activation, as well as inhibition of the TGF‐β/Smad3 pathway. Cell proliferation was also significantly decreased after ARAP1 knockdown. These results suggest that up‐regulated ARAP1 may maintain a high level of total EGFR by reducing the degradation of total EGFR and subsequently promoting high glucose‐induced proximal tubular cell proliferation and fibrosis by maintaining persistent activation of the EGFR/TGF‐β/Smad3 pathway. Additionally, the effect of ARAP1‐AS2 overexpression on fibrosis and cell proliferation was rescued by ARAP1 knockdown and the EGFR tyrosine kinase inhibitor AG1478. Overall, our data demonstrate that ARAP1‐AS2 promotes high glucose‐induced proximal tubular cell proliferation and fibrosis through the EGFR/TGF‐β/Smad3 pathway by interacting with ARAP1.

**Figure 8:**
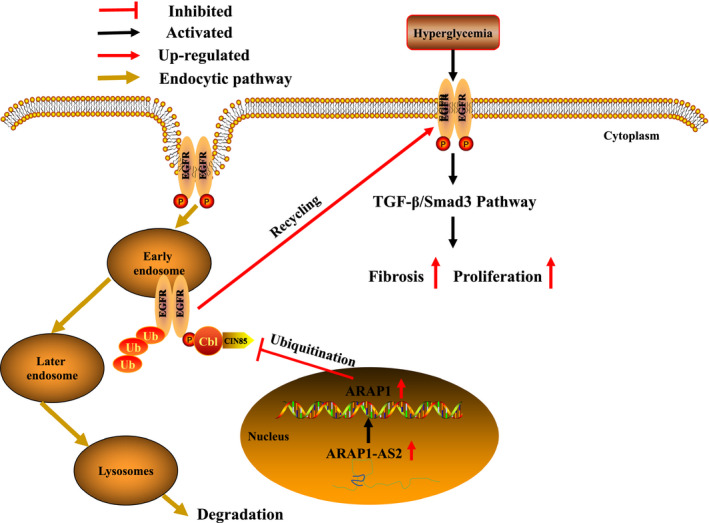
Schematic representation of the proposed model: possible mechanism by which ARAP1‐AS2 is involved in high glucose‐induced proximal tubular cell injury and fibrosis via the EGFR/TGF‐β/Smad3 pathway. Increased ARAP1‐AS2 expression in a high‐glucose environment can up‐regulate ARAP1 expression. Subsequently, ARAP1 competes with Cbl for CIN85 binding in early endosomes and reduces the ubiquitination of EGFR, thus providing more total EGFR proteins to maintain the persistent activation of EGFR in diabetic nephropathy. The persistent activation of EGFR then maintains persistent activation of the TGF‐β/Smad3 pathway and aggravates proximal tubular cell injury and fibrosis in diabetic nephropathy

ARAP1‐AS2 is increased under a high‐glucose environment, which up‐regulated the expression of ARAP1. Subsequently, ARAP1 competes with Cbl for CIN85 binding in early endosomes and reduces the ubiquitination of EGFR, thus providing more total EGFR proteins that can be stimulated by high glucose to maintain the persistent activation of EGFR. Then, the persistent activation of EGFR maintains persistent activation of the TGF‐β/Smad3 pathway under a high‐glucose environment and aggravates proximal tubular cell injury and fibrosis (Figure 8).

In conclusion, this study identified that ARAP1‐AS2 can directly interact with ARAP1 and plays a critical role in high glucose‐induced proximal tubular cell injury and fibrosis by maintaining persistent activation of the EGFR/TGF‐β/Smad3 pathway. The modulation of ARAP1‐AS2 and ARAP1 may provide a novel approach for the treatment of DN.

## CONFLICT OF INTERESTS

The authors declare that they have no conflict of interest.

## AUTHOR CONTRIBUTION

**Xin Li:** Conceptualization (lead); Data curation (lead); Formal analysis (lead); Investigation (lead); Methodology (lead); Resources (lead); Software (lead); Supervision (lead); Validation (lead); Visualization (lead); Writing‐original draft (lead); Writing‐review & editing (lead). **Tian‐Kui Ma:** Conceptualization (supporting); Data curation (supporting); Formal analysis (supporting); Methodology (supporting). **Si Wen:** Data curation (supporting); Formal analysis (supporting); Methodology (supporting). **Lu‐Lu Li:** Formal analysis (supporting); Methodology (supporting); Software (supporting). **Li Xu:** Data curation (supporting); Funding acquisition (supporting); Methodology (supporting). **Xin‐Wang Zhu:** Data curation (supporting); Formal analysis (supporting); Methodology (supporting). **Cong‐Xiao Zhang:** Data curation (supporting); Formal analysis (supporting); Methodology (supporting). **Nan Liu:** Data curation (supporting); Formal analysis (supporting); Methodology (supporting). **Xu Wang:** Conceptualization (supporting); Data curation (supporting); Formal analysis (supporting); Methodology (supporting). **Qiu‐Ling Fan:** Conceptualization (equal); Data curation (supporting); Formal analysis (supporting); Funding acquisition (lead); Methodology (supporting); Writing‐review & editing (equal).

## Supporting information

Appendix S1

## Data Availability

The data generated and analysed during the current study are available from the corresponding author on reasonable request.
