# Supplementary material for: LncRNA ARAP1‐AS2 promotes high glucose‐induced human proximal tubular cell injury via persistent transactivation of the EGFR by interacting with ARAP1
Source: J Cell Mol Med. 2020 Sep 23;24(22):12994–3009. doi: 10.1111/jcmm.15897 (PMC7701572; doi:10.1111/jcmm.15897)
Supplement: Supplementary file 1 — Appendix S1 [file JCMM-24-12994-s001.docx]

| **Gene** | **shRNA sequence** |
| --- | --- |
| ARAP1 | 5′- GCTTCCACGATCGCTACTTCATTCAAGAGATGAAGTAGCGATCGTGGAAGCTTTTTT-3′ |

**Supplementary Table 1. The shRNA sequence targeting ARAP1**

**Supplementary Table 2.** **The siRNA sequence targeting ARAP1-AS2**

| **Gene** | **siRNA sequence** |
| --- | --- |
| ARAP1-AS2 | 5′-GCAAAGUCCUUAGCACUUATT-3′ |

**Supplementary Table 3.** **Gene-specific primers (GSP) of RACE used for PCR**

| **GSP** | **Primer sequence** |
| --- | --- |
| 5′ RACE GSP1 | 5′-GCATGGCCAGAACTTTTCACATGGCTTAG-3′ |
| 5′ RACE GSP2 | 5′-CCTGAGCTGGTAGATGAGATGGGTTAAGACA-3′ |
| 3′ RACE GSP1 | 5′-CCTGTTCCTCATGCTGTCTTAACCCATCTCA-3′ |
| 3′ RACE GSP2 | 5′-CAGCTCAGGGAGATGTCTTGGCATATGG-3′ |

**Supplementary Table 4.** **Primer sequences of qRT-PCR**

| **Gene** | **Primer sequence** |
| --- | --- |
| ARAP1-AS2 | Reverse: GTGGAATGAGGAGCCGAATGAAGG  Forward: GTGCCTAACCTGTCAGCCAATGG |
| ARAP1 | Reverse: CAGCCAGCCAGCCTTGATGAC  Forward: CATACCAGCAGCCTGAGCTTGTC |
| EGFR | Reverse: GAATTCGATGATCAACTCACGG  Forward: ACCCATATGTACCATCGATGTC |
| TGF-β1 | Reverse: TCCAGACTCCAAATGTAG  Forward: GGACACCAACTATTGCTTCAG |
| Smad3 | Reverse: GAAGTTAGTGTTTTCGGGGATG  Forward: AGAGAGTAGAGACACCAGTTCT |
| β-actin | Reverse: CGAGCTCTGAGCACTGGAGA  Forward: TGGCGTGTAAAGTCACCACC |

**Supplementary Table 5.** **ARAP1-AS2 FISH probe sequences**

| 5′-TCTTAACCCATCTCATCTAC-3′ |
| --- |
| 5′-TCAGAAAVVAAGTTTCTGCC-3′ |
| 5′-GTGAACTCACATGTACCAGT-3′ |
| 5′-GACACTTGGAGTGTCACATC-3′ |

**Supplementary Table 6.** **RNA pull-down assay probe sequences**

| **Probe** | **Sequences** |
| --- | --- |
| ARAP1-AS2-Sense-1 | 5′-gacaggcctgaggacacatt-3’ |
| ARAP1-AS2-Sense-2 | 5′-ttcctcagtctcctgcaccc-3’ |
| ARAP1-AS2-Sense-3 | 5′-gccctgcctaagccatgtga-3’ |
| ARAP1-AS2-Sense-4 | 5′-tttctgctgccctgttcctc-3’ |
| ARAP1-AS2-Sense-5 | 5′-ggcaagtaccgccgctacca-3’ |
| ARAP1-AS2-Sense-6 | 5′-gcttctgggcagccaacgtg-3’ |
| ARAP1-AS2-Sense-7 | 5′-accaagtttctgcccggcac-3’ |
| ARAP1-AS2-Sense-8 | 5′-catccccagccaccagcagg-3’ |
| ARAP1-AS2-Sense-9 | 5′-caggtgcaaggcctgaggca-3’ |
| ARAP1-AS2-Sense-10 | 5′-ttggctgacaggttaggcac-3’ |
| ARAP1-AS2-Sense-11 | 5′-atgctggatccctgcactca-3’ |
| ARAP1-AS2-Sense-12 | 5′-gtgactccagcaccccatac-3’ |
| ARAP1-AS2-Sense-13 | 5′-agaacaagccaggagttagg-3’ |
| ARAP1-AS2-Sense-14 | 5′-tttctcacctccccacggtg-3’ |
| ARAP1-AS2-Sense-15 | 5′-ggaaactcccaggtacccata-3’ |
| ARAP1-AS2-Sense-16 | 5′-gaggccctcagcctccattt-3’ |
| ARAP1-AS2-Sense-17 | 5′-tctgcctgagattaccctgt-3’ |
| ARAP1-AS2-Sense-18 | 5′-ctatttcgccctttagagaaag-3’ |
| ARAP1-AS2-Antisense | 5′-agagttgttctttcctttcct-3’ |

**Supplementary Table 7.** **The specific antibody dilution ratios of western blot**

| **Antibody** | **Dilution ratio** | **Source** |
| --- | --- | --- |
| anti-ARAP1 | 1:100 | sc-367892, Santa Cruz Biotechnology, USA |
| anti-EGFR | 1:1000 | ab52894, Abcam, USA |
| anti-p-EGFR（Y1173） | 1:1000 | ab32578, Abcam, USA |
| anti-p-EGFR（Y1068） | 1:1000 | ab40815, Abcam, USA |
| anti-TGF-β1 | 1:2000 | 21898-1-AP, Proteintech Co Ltd, USA |
| anti-p-smad3 | 1:2000 | ab52903, Abcam, USA |
| anti-smad3 | 1:2000 | ab40854, Abcam, USA |
| anti-Collagen I | 1:1000 | 14695-1-AP, Proteintech Co Ltd, USA |
| anti-Collagen IV | 1:1000 | 55131-1-AP, Proteintech Co Ltd, USA |
| anti-Fibronectin | 1:1000 | 15613-1-AP, Proteintech Co Ltd, USA |


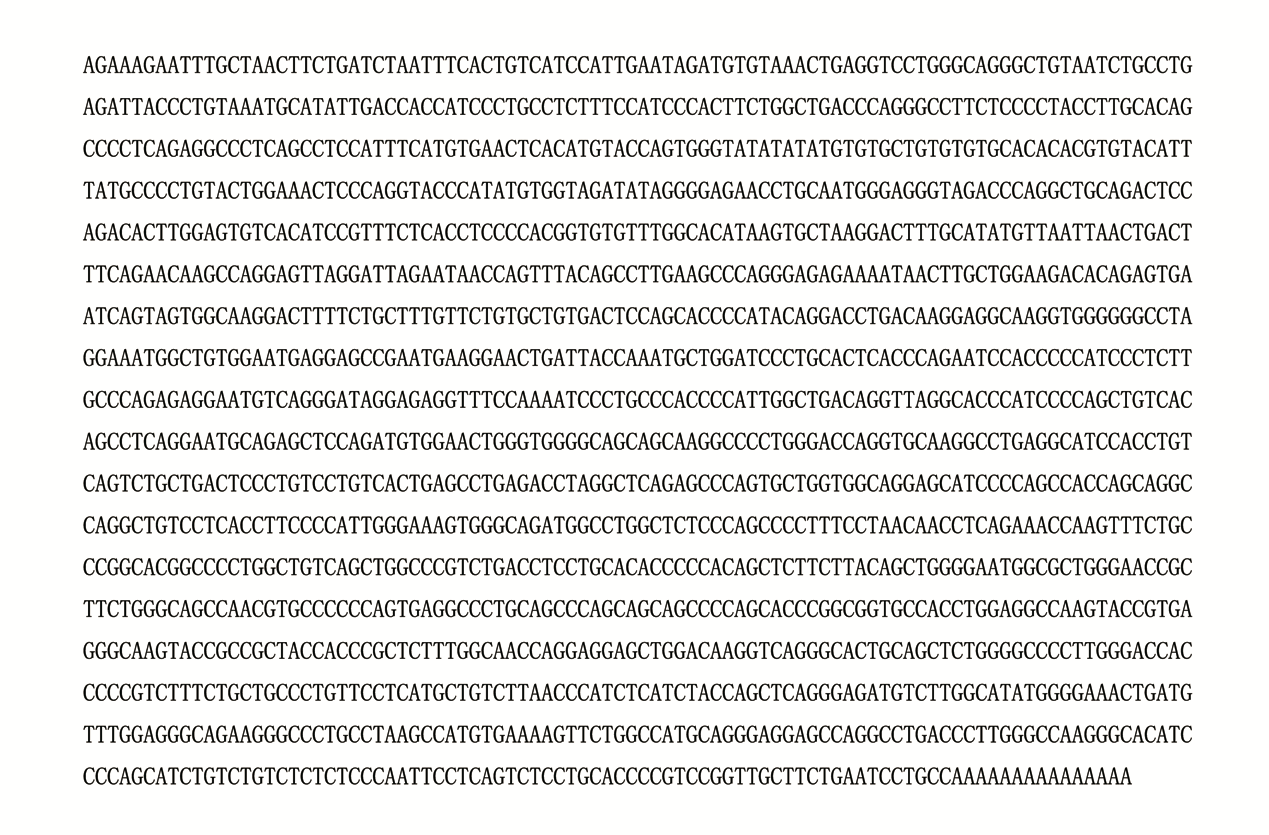


**Figure S1** **The nucleotide sequence of full-length ARAP1-AS2 determined by RACE**


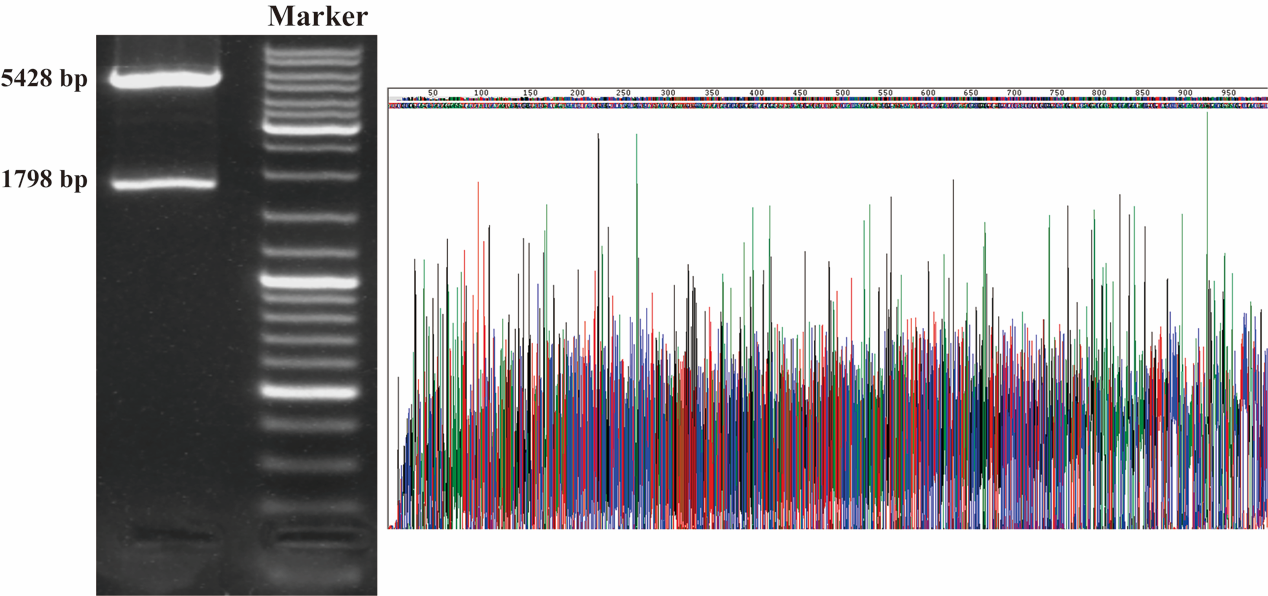


**Figure S2** **Validation of overexpression plasmid of ARAP1-AS2.** ARAP1-AS2 overexpression plasmid was verified by restriction enzyme digestion and nucleotide sequencing.


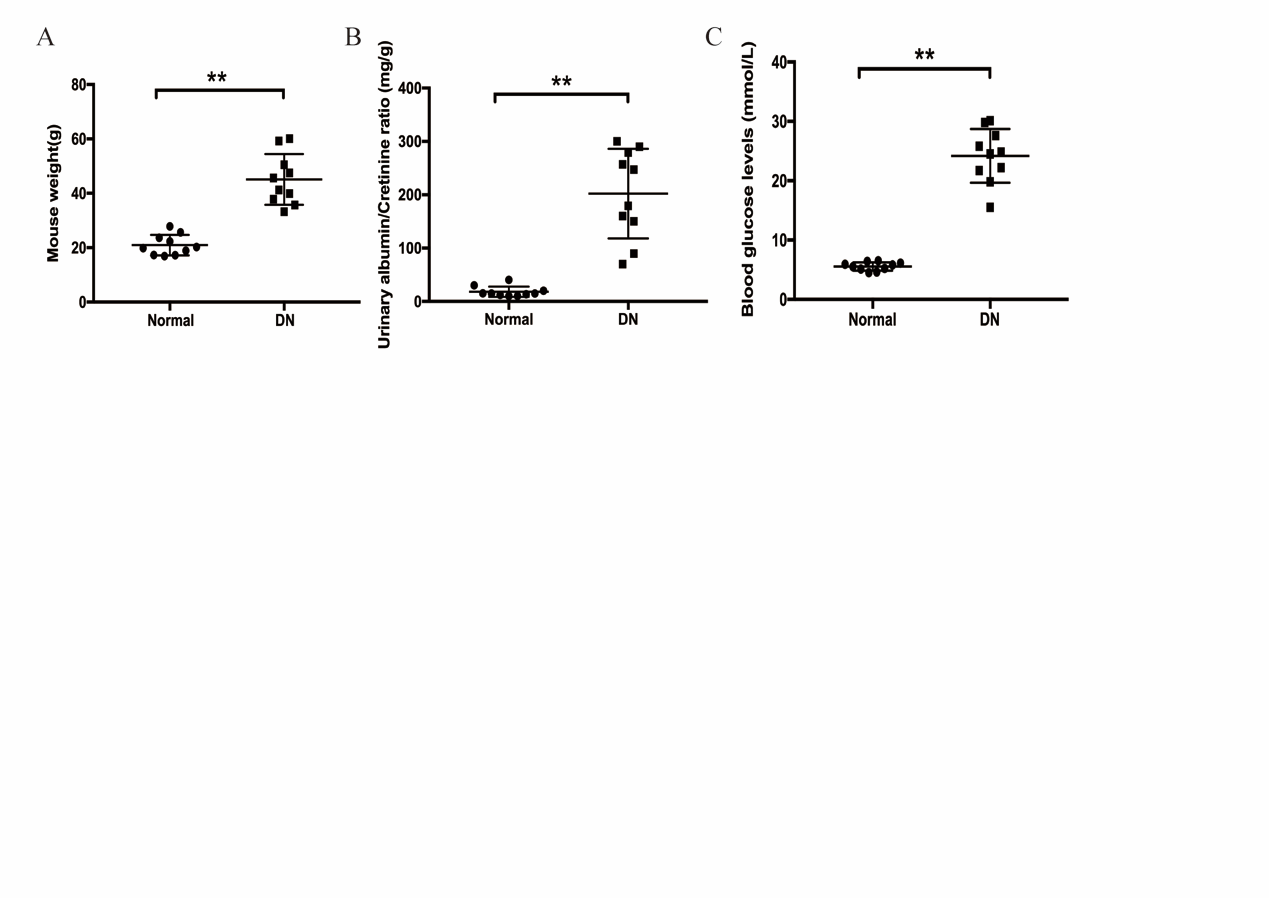


**Figure S3. Body weight, Urinary albumin/Cretinine ratio, and blood glucose of db/db and db/m mice at 20th week.** (A) Body weight (n=10). (B) Urinary albumin/cretinine ratio (n=10). (C) Blood glucose levels (n=10). Data are presented as mean±SD. **P<0.01.
